# Immunogenicity Evaluating of the Multivalent COVID-19 Inactivated Vaccine against the SARS-CoV-2 Variants

**DOI:** 10.3390/vaccines10060956

**Published:** 2022-06-16

**Authors:** Yuntao Zhang, Wenjie Tan, Zhiyong Lou, Baoying Huang, Weimin Zhou, Yuxiu Zhao, Jin Zhang, Hongyang Liang, Na Li, Xiujuan Zhu, Ling Ding, Yancen Guo, Zhenyu He, Yao He, Zhanhui Wang, Bo Ma, Meng Ma, Suhua Zhao, Zhen Chang, Xue Zhao, Xiaotong Zheng, Guizhen Wu, Hui Wang, Xiaoming Yang

**Affiliations:** 1 China National Biotec Group Company Limited, Beijing 100024, China; zhangyuntao@sinopharm.com; 2 National Institute for Viral Disease Control and Prevention, Chinese Center for Disease Control and Prevention (China CDC), Beijing 102206, China; tanwj@ivdc.chinacdc.cn (W.T.); baoying1233@163.com (B.H.); doglet44@163.com (W.Z.); 3 MOE Key Laboratory of Protein Science & Collaborative Innovation Center of Biotherapy, School of Medicine, Tsinghua University, Beijing 100084, China; louzy@mail.tsinghua.edu.cn; 4 Beijing Institute of Biological Products Company Limited, Beijing 100176, China; zhao10306@126.com (Y.Z.); zhangjin_2566@sina.com (J.Z.); ayangyang@163.com (H.L.); lina1@sinopharm.com (N.L.); zhuxiujuan1@sinopharm.com (X.Z.); dinglingmail@163.com (L.D.); nancyguo1112@sina.com (Y.G.); zhenyu_he09@126.com (Z.H.); heyao142021@163.com (Y.H.); wangzhanhui2016@163.com (Z.W.); mabo0303@163.com (B.M.); kelemeng215@139.com (M.M.); baiyueguangfeifei@163.com (S.Z.); czhen9008@163.com (Z.C.); xiaoxueer012.student@sina.com (X.Z.); zhengxiaotong20@sina.com (X.Z.)

**Keywords:** Beta, Delta, HB02, immunogenicity, multivalent inactivated vaccines, Omicron, SARS-CoV-2

## Abstract

It has been reported that the novel coronavirus (COVID-19) has caused more than 286 million cases and 5.4 million deaths to date. Several strategies have been implemented globally, such as social distancing and the development of the vaccines. Several severe acute respiratory syndrome coronavirus 2 (SARS-CoV-2) variants have appeared, such as Alpha, Beta, Gamma, Delta, and Omicron. With the rapid spread of the novel coronavirus and the rapidly changing mutants, the development of a broad-spectrum multivalent vaccine is considered to be the most effective way to defend against the constantly mutating virus. Here, we evaluated the immunogenicity of the multivalent COVID-19 inactivated vaccine. Mice were immunized by multivalent COVID-19 inactivated vaccine, and the neutralizing antibodies in serum were analyzed. The results show that HB02 + Delta + Omicron trivalent vaccine could provide broad spectrum protection against HB02, Beta, Delta, and Omicron virus. Additionally, the different multivalent COVID-19 inactivated vaccines could enhance cellular immunity. Together, our findings suggest that the multivalent COVID-19 inactivated vaccine can provide broad spectrum protection against HB02 and other virus variants in humoral and cellular immunity, providing new ideas for the development of a broad-spectrum COVID-19 vaccine.

## 1. Introduction

The COVID-19 outbreak began in December 2019 and spread rapidly, threatening the lives of people around the world. As of March 2022, the SARS-CoV-2 virus has infected 345 million people worldwide (www.who.int (March 2022)). Vaccines have effectively reduced the infection rate and mortality rate, but with the emergence of virus variants, there have been many reports on the reduction in neutralization titers in vaccinees’ serum against different variants. Reduced neutralization has been shown for several approved vaccines, such as mRNA1273, ChAdOx1, BNT162b2, nCoV-19, and NVX-CoV2373 [1]. There are currently five variants of concern (VOC) announced by the World Health Organization (WHO), namely Alpha (B.1.1.7), Beta (B.1.351), Gamma (P.1), Delta (B.1.617.2), and Omicron (B.1.1.529) [2]. Many of their amino acid mutations in the receptor-binding domain (RBD) region of spike proteins have been reported to be closely related to immune escape. For example, Beta harbors mutations in E484 and N501 that diminish vaccine efficiency [3], and the neutralizing level against the Beta variant in BNT162b2- and AZD1222-vaccinated serum was lower [4].

Due to the rapid mutation rate, vaccines may lose effectiveness against COVID-19 variants. In fact, it has been reported that recombinant trimeric RBD [5] and neutralizing antibodies [6] have neutralizing effects against the Beta and Delta variants, not Omicron, and broad protection against the Omicron variant has not yet been reported. Therefore, there is an urgent need to develop a broad-spectrum vaccine against the different COVID-19 variants. Both B cell antibody-mediated humoral immunity and T-cell-mediated cellular immunity are necessary for an effective vaccine [7]. Here, we test the immunogenicity of a multivalent COVID-19 inactivated vaccine against the HB02, Beta, Delta, and Omicron variants, and found that the HB02 + Delta + Omicron trivalent vaccine could provide broad spectrum protection against HB02 and other virus variants in humoral and cellular immunity.

## 2. Results

**Figure 1:**
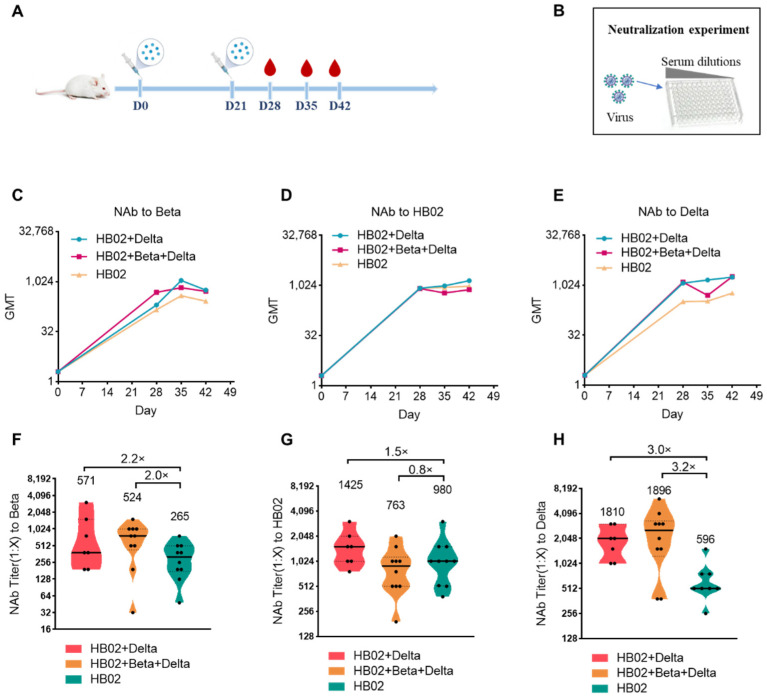
The neutralizing antibody titers of the HB02, HB02 + Delta, and HB02 + Beta + Delta vaccines against different variants. (**A**) Schematic diagram of the immunization program—the BALB/c mice were injected with different vaccines on day 0 and day 21, and blood was collected on days 28/35/42 after immunization. (**B**) The neutralizing antibody titers (NAb Titer) at 28/35/42 days after the administration of the vaccine against different variants were tested by means of the microplate micro-cytopathogenic efficiency (CPE) method. (**C**) The NAb titers of the HB02, HB02 + Delta, and HB02 + Beta + Delta vaccines against the Beta variant on days 0/28/35/42 were obtained by means of the microtitration method (*n* = 7–10). (**D**) The Nab titers of the HB02, HB02 + Delta, and HB02 + Beta + Delta vaccines against the HB02 variant on days 0/28/35/42 were obtained by means of the microtitration method (*n* = 7–10). (**E**) The NAb titers of the HB02, HB02 + Delta, and HB02 + Beta + Delta vaccines against the Delta variant on days 0/28/35/42 were obtained by means of the microtitration method (*n* = 7–10). (**F**) The NAb titers of the HB02, HB02 + Delta, and HB02 + Beta + Delta vaccines against the Beta variant on day 42 were obtained by means of the microtitration method (*n* = 7–10). (**G**) The NAb titers of the HB02, HB02 + Delta, and HB02 + Beta + Delta vaccines against the HB02 variant on day 42 were obtained by means of the microtitration method (*n* = 7–10). (**H**) The NAb titers of the HB02, HB02 + Delta, and HB02 + Beta + Delta vaccines against the Delta variant on day 42 were obtained by means of the microtitration method (*n* = 7–10).

**Figure 2:**
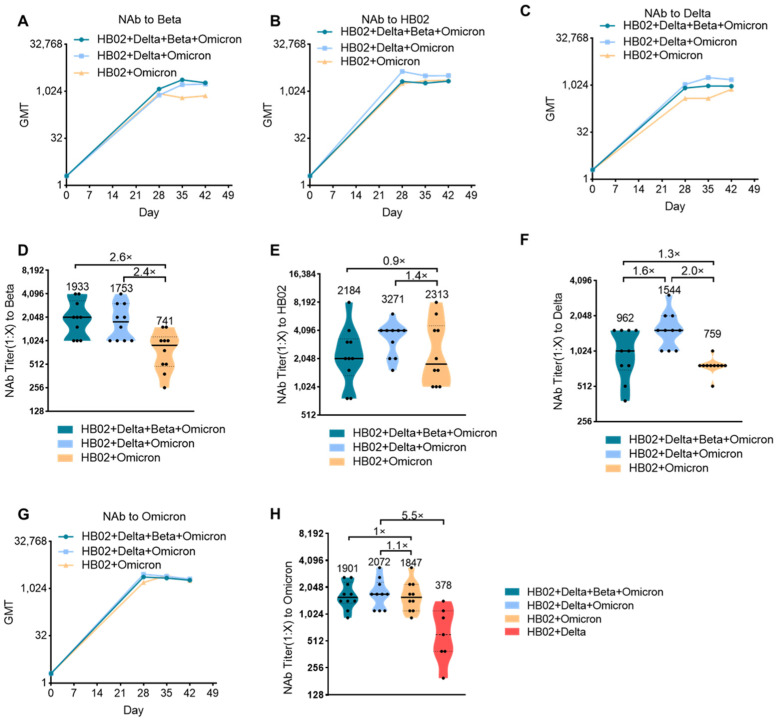
Comparison of the broad protection ability of the Omicron + Delta + Beta + HB02, Delta + Beta + HB02, and HB02 + Omicron vaccines. (**A**) The GMTs of Omicron + Delta + Beta + HB02, Delta + Beta + HB02, and HB02 + Omicron vaccines against the Beta variant on days 0/28/35/42 were obtained by means of the microtitration method (*n* = 10). (**B**) The GMTs of the Omicron + Delta + Beta + HB02, Delta + Beta + HB02, and HB02 + Omicron vaccines against the HB02 variant on days 0/28/35/42 were obtained by means of the microtitration method (*n* = 7–10). (**C**) The GMTs of the Omicron + Delta + Beta + HB02, Delta + Beta + HB02, and HB02 + Omicron vaccines against the Delta variant on days 0/28/35/42 were obtained by means of the microtitration method (*n* = 10). (**D**) The NAb titers of the Omicron + Delta + Beta + HB02, Delta + Beta + HB02, and HB02 + Omicron vaccines against the Beta variant on day 42 were obtained by means of the microtitration method (*n* = 10). (**E**) The NAb titers of the Omicron + Delta + Beta + HB02, Delta + Beta + HB02, and HB02 + Omicron vaccines against the HB02 variant on day 42 were obtained by means of the microtitration method (*n* = 10). (**F**) The NAb titers of the Omicron + Delta + Beta + HB02, Delta + Beta + HB02, and HB02 + Omicron vaccines against the Delta variant on day 42 were obtained by means of the microtitration method (*n* = 10). (**G**) The GMTs of the Omicron + Delta + Beta + HB02, Delta + Beta + HB02, and HB02 + Omicron vaccines against the Omicron variant on days 0/28/35/42 were obtained by means of the microtitration method (*n* = 10). (**H**) The NAb titers of the Omicron + Delta + Beta + HB02, Delta + Beta + HB02, and HB02 + Omicron vaccines against the Omicron variant on day 42 were obtained by means of the microtitration method (*n* = 7–10).

The emergence of the variant strains of SARS-CoV-2 increased the risk of infection. So far, compared with the ancestral virus, the reported VOCs Beta (B.1.351), Delta (B.1.617.2), and Omicron (B.1.1.529) have some mutations in the RBD and the N-terminal domain (NTD), which caused the variants to evade current vaccines. To explore the best immunization strategy, we first used a multivalent COVID-19 inactivated vaccine including the bivalent vaccine based on the original strain and Delta variant (HB02 + Delta), and a trivalent vaccine based on the original strain and Delta and Beta variants (HB02 + Beta + Delta). BALB/c mice were injected with different vaccines (HB02, HB02 + Delta, HB02 + Beta + Delta) at day 0 and day 21, and the NAb titers at 28/35/42 days after the administration of the vaccine against different variants were tested (Figure 1A,B). The results show that the neutralization geometric mean titers (GMTs) against different variants after the administration of the vaccine rose over time (Figure 2C–E). However, comparing the neutralization effects of different vaccines against variant strains, the GMTs of the serum from mice immunized with the HB02 + Delta vaccine against Beta, HB02, and Delta were 571, 1425, and 1810, respectively, which were 2.2-, 1.5-, and 3.0-fold higher those of the HB02 vaccine (GMT = 265, 980, and 596, respectively), and the GMTs of the serum from mice immunized with the HB02 + Beta + Delta vaccine against Beta, HB02, Delta, and Omicron were 524, 763, and 1896, respectively, which were 2.0-, 0.8-, and 3.2-fold higher than those of the HB02 vaccine (GMT = 265, 980, and 596, respectively) (Figure 1F–H). This indicated that the HB02 + Delta and HB02 + Beta + Delta vaccines provide effective protection against different variants. Then, compared with the HB02 + Beta + Delta vaccine, the neutralizing ability of the HB02 + Delta vaccine against the Beta and Delta virus was comparable. However, the neutralizing ability of the HB02 + Delta vaccine against the HB02 virus was higher than that of the HB02 + Beta + Delta vaccine. This indicates that the HB02 + Delta bivalent vaccine could provide effective protection against different variants.

The impact of the Delta variant on society has not faded, and the sudden appearance of the Omicron variant has once again surpassed people’s expectations regarding the development of the novel coronavirus and the pandemic. Against the background of global COVID-19 vaccinations exceeding 10 billion doses, people who have received two doses of existing vaccines will still experience breakthrough infection cases with Omicron, which has exceeded the impact of Delta and has become the world’s major pandemic variant and led to a rebound of the epidemic in many countries and regions. Therefore, the development of a new generation of vaccines with sufficient protection against mutant strains including the Omicron mutant has become an urgent need. Although the bivalent HB02 + Delta vaccine had a protective effect on different strains (HB02, Beta, and Delta), the protective effect against the Omicron variant is not clear. Next, we tested the protective effect of the HB02 + Delta bivalent vaccine against the Omicron variant. The results show that the GMT level of the neutralizing antibody against Omicron after 42 days for the HB02 + Delta bivalent vaccine was about 378, demonstrating weak protection against the Omicron variant (Figure 2H). To improve the protective effect of the vaccine, we used a multivalent COVID-19 inactivated vaccine including a bivalent vaccine based on the original strain and the Omicron variant (HB02 + Omicron), and a trivalent vaccine based on the original strain and the Delta and Omicron variants (HB02 + Delta + Omicron), and a tetravalent vaccine based on the original strain and the Delta, Beta, and Omicron variants (HB02 + Delta + Beta + Omicron). BALB/c mice were injected with different vaccines (HB02 + Omicron, HB02 + Delta + Omicron, HB02 + Beta + Delta + Omicron) on day 0 and day 21, and the neutralizing antibody titers (NAb Titer) at 28/35/42 days after the administration of the vaccine against different variants were tested. The results show that the neutralization GMTs against different variants after the administration of the vaccine rose over time (Figure 2A–C,G). The GMTs of the serum of mice immunized with the HB02 + Omicron vaccine against Beta, HB02, Delta, and Omicron were 741, 2313, 759, and 1847, respectively, the GMTs of the serum of mice immunized with the HB02 + Delta + Omicron vaccine against Beta, HB02, Delta, and Omicron were 1753, 3271, 1544, and 2072, respectively, and the GMTs of the serum of mice immunized with the HB02 + Beta + Delta + Omicron vaccine were 1933, 2184, 962, and 1901, respectively. Compared with the GMTs of the serum of mice immunized with the HB02 + Beta + Delta + Omicron vaccine, it was found that the HB02 + Delta + Omicron trivalent vaccine could provide effective protection against different variants. Specifically, the GMT against Delta was 1544, significantly increased by 1.6-fold compared to the HB02 + Delta + Omicron trivalent vaccine group, (Figure 2F), and the GMTs against other viruses displayed no significant differences in these two groups (Figure 2D–F,H). Additionally, compared with the bivalent vaccine, the increases in GMT against Beta (Figure 2D), HB02 (Figure 2E), Delta (Figure 2F), and Omicron (Figure 2H) were 2.4-, 1.4-, 2.0-, and 1.1-fold in the HB02 + Delta + Omicron trivalent vaccine group. This indicates that the HB02 + Delta + Omicron trivalent vaccine could provide effective protection against different variants. In order to detect which vaccines were better in a broad spectrum, we compared the HB02 + Delta bivalent vaccine and HB02 + Delta + Omicron trivalent vaccine and found that the GMT of the HB02 + Delta + Omicron trivalent vaccine against the Omicron variant was 2072, which was higher (5.5-fold) than that of the HB02 + Delta bivalent vaccine. Here, we show that the HB02 + Delta + Omicron trivalent vaccine was effective enough against different variants.

**Figure 3:**
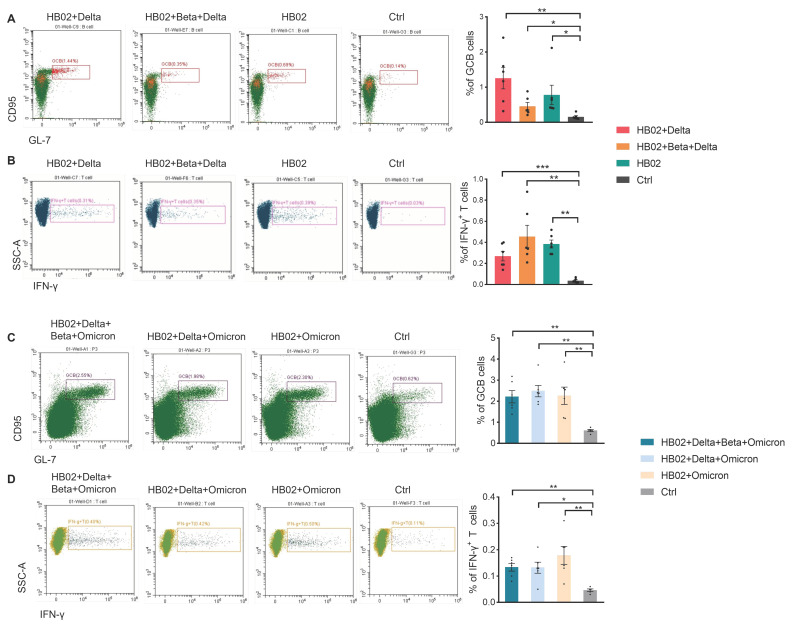
Cellular response of splenocytes in mice immunized with different vaccines. (**A**,**C**) The percentage of GCB cells was analyzed by means of flow cytometry in mice immunized with the HB02 + Delta, HB02 + Beta + Delta, and HB02 vaccines or the control group after 42 days (*n* = 5–6), with GCB cells gated in live CD45 + B220 +; (**B**,**D**) The percentage of IFN-γ-expressing T cells was analyzed by means of flow cytometry in mice immunized with HB02 + Delta, HB02 + Beta + Delta, and HB02 vaccines or the control group after 42 days (*n* = 5–6), with IFN-γ-expressing T cell gated in live CD45 + CD90 +. Error bars represent SEM. * *p* < 0.05; ** *p* < 0.01; *** *p* < 0.001 ((**A**–**D**), Mann–Whitney test). See also Figure S1.

**Figure 4:**
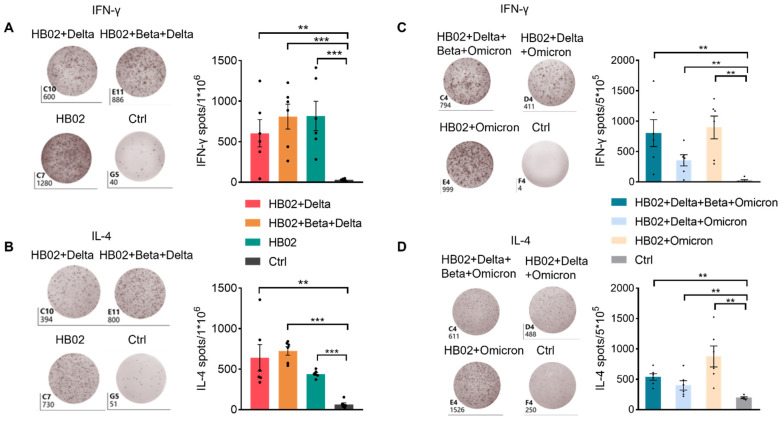
Cytokine response of splenocytes in mice immunized with different vaccines. Mice were immunized with different vaccines, and splenocytes were collected on day 42 post immunization. (**A**,**B**) Virus-specific IFN-γ and IL-4 in spleens were measured by means of the ELISPOT assay (*n* = 5–6) after injection of the HB02 + Delta, HB02 + Beta + Delta, and HB02 vaccines or the control group. (**C**,**D**) Virus-specific IFN-γ and IL-4 in spleens were measured by means of the ELISPOT assay (*n* = 5–6) after injection of the HB02 + Omicron, HB02 + Omicron + Delta, and HB02 + Omicron + Delta + Beta vaccines or the control group. Error bars represent SEM. ** *p* < 0.01; *** *p* < 0.001 ((**A**–**D**), Mann–Whitney test).

In addition to neutralizing antibodies, the body also relies on CD8+ T cells and CD4+ T cells to clear viruses. To date, many studies have focused on the T cells [8]. Several studies have successfully isolated T cells that attack the novel coronavirus from the blood of patients who had recovered a long time ago. When stimulated by the novel coronavirus, these T cells could replicate themselves and release signals to fight the virus [9]. Additionally, the CD8^+^ T cells play an important role in fighting 2019-nCoV and could form long-term immune responses, and some vaccines have been reported to have the ability to promote cellular immune responses, such as the Pfizer and Moderna vaccines [10,11]. Recently, some papers showed that the T cells obtained from vaccine recipients could significantly recognize various mutant strains and produce cytokines to play an important role. Therefore, most mutant epitopes of mutant strains could still be recognized by T cells, and then perform important functions. Here, we also tested the effect of a multivalent COVID-19 inactivated vaccine on humoral and cellular immunity. We isolated the immune cells from mouse spleens immunized with the different vaccines and analyzed the total B cells and T cells. There were no significant differences in the abundance of total B and T cells post administration of different vaccines (Figure S1). Since the germinal center B cells (GCB) are the source of the high-affinity antibodies required for protective immunity [12], we analyzed GCB cells in the spleen after vaccine immunization. The results show that, compared with the control group, the percentage of GCB cells in different groups was significantly increased (Figure 3A,C), suggesting that vaccines induce a strong humoral immune response. Previous studies showed that IL-4 belongs to the Th2 cytokine, which can regulate antibody class switching. Additionally, mRNA vaccines have been reported to be able to induce SARS-CoV-2-specific T-cell responses, especially IFN-γ production [13,14]. Here, we measured IFN-γ and IL-4 to evaluate the humoral and cellular immunity. In addition to B cells, we also found that compared with the control group, the secretion of IFN-γ in T cells or other cells was also induced after the different vaccine immunizations (Figure 3B,D and Figure S2). Additionally, this suggests that the multivalent COVID-19 inactivated vaccine could induce humoral and cellular immune responses. To confirm this, we also analyzed the secretion of IL-4 and IFN-γ by ELISPOT. Additionally, consistent with FACS data, IL-4 and IFN-γ were elevated after two dose immunization with the different vaccines (Figure 4A–D). This proves that the inactivated vaccine could effectively promote the humoral immune response and the cellular immune response after two dose immunization, which demonstrated that our inactivated vaccine had a strong protective effect against the virus.

## 3. Discussion

SARS-CoV-2 has spread globally since December 2019. The COVID-19 pandemic caused by SARS-CoV-2 represents a serious threat to global public health and the economy. As an RNA virus, SARS-CoV-2 will inevitably mutate over the course of the pandemic. The WHO classifies important variants into variants of concern (VOI) and VOC according to their prevalence. There are currently five variants, namely Alpha, Beta, Gamma, Delta, and Omicron. Owing to the many key mutation sites in the spike protein (S protein) of the virus variants, there are concerns that those variants will largely evade vaccine-elicited immunity [4]. Additionally, it has been reported that variants had a large number of mutations, especially in the RBD of S protein, which resulted in significantly decreased protection against the variant being provided by existing vaccines. The neutralizing antibody levels of the serum immunized with the Pfizer BNT162b2 vaccine (Pfizer-BioNTech, New York, NY, USA) against Omicron decreased by 41-fold compared with the prototype strain [15]. Another paper reported that there was a 22-fold reduction in Pfizer BNT162b2 vaccine-induced neutralization against the Omicron variant [16]. Additionally, for the mRNA-1273 (Spikevax, Cambridge, MA, USA) vaccine, there was a 20-fold reduction compared to D614G [17]. Additionally, the neutralization potency of the serum from mRNA-1273, BNT162b, and Ad26.COV2.S vaccine recipients against wild-type, Delta, and Omicron SARS-CoV-2 pseudoviruses was decreased [18]. Additionally, there are many ongoing vaccine and therapeutic trials, such as monoclonal antibodies, small molecules, plasma therapy, etc., designed to contain the COVID-19 pandemic [19,20]. In addition, we urgently need to develop a broad-spectrum vaccine. Here, we use the multivalent COVID-19 inactivated vaccine to immunized mice and compared the immunogenicity of the multivalent COVID-19 inactivated vaccine against the SARS-CoV-2 variants. Our study proves that HB02 + Delta + Omicron trivalent vaccine conferred highly efficient protection against different virus variants in mice.

Neutralizing antibodies are responsible for the removal of extracellular viruses and prevent the virus from infecting host cells. In the early days of the fight against SARS-CoV-2, the vast majority of vaccine research and development efforts focused on neutralizing antibodies produced by B cells. However, neutralizing antibodies in most SARS-CoV-2 survivors dropped to baseline levels within a few months, suggesting that a generation of vaccines that induce humoral immunity may not be sufficient for long-term immunity against SARS-CoV-2. The final clearance of the virus in infected organ cells requires a cellular immune response involving T cells [21]. Additionally, except for neutralizing antibodies, there are many papers that reported that immune memory can be achieved through virus-specific memory T cells [22], and mRNA vaccines could elicit strong T-cell responses [10,23]. In addition, T cells play an important role in fighting the novel coronavirus and forming a long-term immune response. Given those studies, we asked whether our multivalent COVID-19 inactivated vaccine would enhance T-cell responses to SARS-CoV-2. We performed FACS and ELISPOT assays on spleen immune cells after vaccination to test the immune response of the virus-specific T cells. Interestingly, both the CD4^+^ T and CD8^+^ T cells from multivalent COVID-19 inactivated vaccine-immunized mice could secrete more IFN-γ after viral stimulation. This indicated that multivalent COVID-19 inactivated vaccine could induce high-efficiency neutralizing antibodies and enhance the activation effect of cellular immunity. The major discovery that inactivated vaccines could induce cellular immune responses seems to open the door to the link between inactivated vaccines and cellular immunity. This paper clarifies that cellular immune responses can work in inactivated vaccines, which helps us to understand that it will be more helpful for the development of inactivated vaccines. However, the mechanism by which inactivated vaccines induce cellular immune responses remains to be studied.

From the perspective of different types/variations of pathogens, it is the most mature choice to develop multivalent vaccines, such as 23-valent polysaccharide vaccine, quadrivalent meningitis Vaccine, 2/4/9-valent HPV vaccine, and 5-valent rotavirus vaccine. SARS-CoV-2 may have spread faster, sooner than previously known, and is highly susceptible to mutations to produce new strains. The development of a multivalent vaccine against SARS-CoV-2 could help to elicit potent broadly neutralizing antibodies. This exploration could serve as an instructive guide for multivalent vaccine development.

Taken together, our results show that the HB02 + Delta + Omicron trivalent vaccine could provide broad-spectrum protection not just against HB02, but against some other virus variants as well. Our data also suggest that vaccine-elicited T cells could effectively secrete IFN-γ after SARS-CoV-2 variants’ stimulation, which could provide protection from SARS-CoV-2 variants, and the mechanism of inactivated vaccines to induce cellular immune responses remains to be studied in future.

Limitations of Study: The major limitation of the present study is the experimental animal model, as we only evaluated the multivalent vaccine-induced humoral and cellular immunity in mice, while the protective efficacy of the vaccine in mice was not evaluated due to the insusceptibility of SARS-CoV-2 in mice. 

### 3.1. Method Details

#### 3.1.1. Animal Models

Mice were purchased from Beijing Weitonglihua Experimental Animal Technology Co., Ltd., Beijing, China. All of the mice in this study were healthy and were provided with a 12 h light/dark cycle (temperature: 18–28 °C, humidity: 40–70%). The mice were maintained in a specific pathogen-free (SPF) environment at the Laboratory Animal Center of Beijing Institute of Biological Products Co., Ltd., Beijing, China. All of the mice were BALB/c, different vaccines, and weighed 17–19 g. The total number of animals per group (as well as each sex per group) is presented in the Table 1.

#### 3.1.2. Reagent

The reagents’ information is presented in the Table 2.

#### 3.1.3. Vaccine Preparation

The monovalent vaccine was prepared as described previously [1]. To inactivate the virus, β-propionolactone was mixed with the harvested viral solution at a ratio of 1:4000 at 4–8 °C. After 20–24 h, the inactivated virus was purified. The multivalent vaccine was prepared by mixing the aluminum adjuvant with the final purified virus at a specified ratio.

#### 3.1.4. Vaccine Immunogenicity Analysis and Neutralization Assay

Female and male mice were divided into different groups and immunized with different vaccines (0.5 mL/mice) intramuscularly on D0 and D21. Blood was collected on D28, D35, and D42, and the spleens were collected on D42. The neutralization assay was based on the microplate CPE (micro-cytopathogenic efficiency) method. Briefly, the serum was diluted by a 2-fold series, starting with a dilution ratio of 1:4, and then the virus was added to the plate and incubated for 2 h in a 37 °C incubator to initiate the neutralization. The cell suspension was added and incubated for another 4 days, followed by observing the CPE. Here, HB02 was the prototype strain, Beta was B.1.351, Delta was B.1.617.2, and Omicron was BA.1. The detailed protocol was described by [1].

#### 3.1.5. Flow Cytometry

For lymphocyte analysis in spleens, the spleens were minced and ground with an injection syringe, and then lysed with red blood cells using RBC (Biolegend, San Diego, CA, USA, 420301). Briefly, 1 mL of RBC Lysis Buffer was added to each sample. After 10 min, 5 mL of PBS was added to the sample, which stopped lysis, and then the samples were centrifuged for 5 min, and we discarded the supernatant and washed the samples two times. The cells were filtered before use. For surface staining, cells were blocked with CD16/32 antibody in FACS buffer (PBS + 0.1% FBS + 0.1% NaN_3_) for 30 min at 4–8 °C, then the cells were stained with antibodies against surface antigens in blocking buffer on ice for 30 min using appropriate antibodies. For IFN-γ staining, immune cells were seeded on 96-well plates at a density of 1 × 10^6^ and were stimulated with inactivated virus stock solution (8 μg/well) in DMEM medium (Gibco, Thermo Fisher Scientific, Waltham, MA, USA, C11995500BT) with 1% penicillin-streptomycin (Procell, PB180120) and 10% FBS (Gibco, Thermo Fisher Scientific, Waltham, MA, USA) for 10 h, and BFA (Biolegend, San Diego, CA, USA, 420601) for another 4 h, and after surface staining, were fixed with IC Fixation Buffer (eBioscience, San Jose, CA, USA). Dead cells were excluded by Fixable Viability Dye eFluor 506 (PBS). Flow cytometry was performed on CytoFLEX S instruments (Beckman, Brea, CA, USA). All of the cells were gated in live cells, and T cells (CD45+ CD90+), CD4+ T cells (CD45+ CD90+ CD4+), CD8+ T cells (CD45+ CD90+ CD4-), and GCB cells (CD45+ B220+GL-7+CD95+) were analyzed with CytoFLEX S FlowJo software. Antibodies were purchased from BioLegend, eBioscience, or BD. The dosages of the antibodies are shown in the Table 3.

#### 3.1.6. ELISPOT Assay

The IFN-γ and IL-4 production was determined with the Mouse IFN-γ precoated ELISPOT kit and the Mouse IL-4 precoated ELISPOT kit according to the manufacturer’s protocol (Dakewe Group, Shenzhen, China). Briefly, the spleens were removed, and the immune cells were purified. In total, 1 × 10^6^ or 5 × 10^5^ immune cells were incubated with virus stock solution (8 μg/well) for 36 h. The spots were counted using a ELIspot Reader System.

#### 3.1.7. Statistics

All statistical analyses were performed by GraphPad Prism 8. In comparisons between the two groups, a two-tailed Mann–Whitney test was used to determine significance. Error bars represent SEM. * *p* < 0.05; ** *p* < 0.01; *** *p* < 0.001.

## Figures and Tables

**Table 1:** Total number of animals per group.

Experiment	Group	Total Number	Male	Female
Neutralization Analysis	HB02 + Delta	7	4	3
	HB02 + Beta + Delta	10	5	5
	HB02	10	5	5
Immunoassay	HB02 + Delta	6	3	3
	HB02 + Beta + Delta	6	3	3
	HB02	6	3	3
Neutralization Analysis	HB02 + Delta + Beta + Omicron	10	5	5
	HB02 + Delta + Omicron	10	5	5
	HB02 + Omicron	10	5	5
Immunoassay	HB02 + Delta + Beta + Omicron	6	3	3
	HB02 + Delta + Omicron	6	3	3
	HB02 + Omicron	6	3	3

**Table 2:** The information of the reagent.

Reagent	Source	CAT#
Purified anti-mouse CD16/32 Antibody	BioLegend (San Diego, CA, USA)	101302
PE/Cyanine7 anti-mouse CD3ε Antibody	BioLegend (San Diego, CA, USA)	100320
FITC anti-mouse CD90.2 Antibody	BioLegend (San Diego, CA, USA)	105306
AF700 anti-mouse/human CD45R/B220 Antibody	BioLegend (San Diego, CA, USA)	103232
Super Bright 600 anti-mouse CD4 Antibody	ThermoFisher (Waltham, MA, USA)	63-0041-82
Fixable Viability Dye eFluor™ 506	ThermoFisher (Waltham, MA, USA)	65-0865-14
FITC anti-mouse GL-7 Antibody	Biolegend (San Diego, CA, USA)	144603
APC anti-mouse IFN-γ Antibody	Ebioscience (Thermo Fisher Scientific, Waltham, MA, USA)	17-7311-82
PE anti-mouse CD95 Antibody	BD (San Jose, CA, USA)	561985
IL-4 ELISPOT	Dakewe Group (Shenzhen, China)	2210402
IFN-g ELISPOT	Dakewe Group (Shenzhen, China)	2210005
BD Cytofix/Cytoperm Fixation/Permeabilization Solution Kit	BD (San Jose, CA, USA)	554714

**Table 3:** The dosage of the reagent.

Reagent	Dosage
Purified anti-mouse CD16/32 Antibody	0.125 μg/50 μL
PE/Cyanine7 anti-mouse CD3ε Antibody	0.05 μg/50 μL
FITC anti-mouse CD90.2 Antibody	0.05 μg/50 μL
AF700 anti-mouse/human CD45R/B220 Antibody	0.05 μg/50 μL
Super Bright 600 anti-mouse CD4 Antibody	0.05 μg/50 μL
Fixable Viability Dye eFluor™ 506	1000×
FITC anti-mouse GL-7 Antibody	0.1 μg/50 μL
APC anti-mouse IFN-γ Antibody	0.15 μg/50 μL
PE anti-mouse CD95 Antibody	0.1 μg/50 μL

## Data Availability

The data presented in this study are available within the article and supplementary materials.

## Supplementary Materials

The following supporting information can be downloaded at: https://www.mdpi.com/article/10.3390/vaccines10060956/s1, Figure S1: The effect of vaccines on various immune cells. (A) The representative flow cytometry figures of B cells (gated in live CD45+), T cells (gated in live CD45+ CD90+), CD8+ T cells (gated in live CD45+ CD90+ CD4-) and CD4+ T cells (gated in live CD45+ CD90+ CD4) in spleens are shown. (B) Percentages of B cells, T cells, CD4+ T and CD8+ T cells in the spleen were analyzed using flow cytometry.; Figure S2: IFN-γ-expressing cell analysis by FACS. (A) The representative flow cytometry figures of IFN-γ+ T cells (gated in live CD45+) in spleens are shown. (B) Percentages of CD90-IFN-γ+ cells in the spleen were analyzed using flow cytometry. Error bars represent SEM. * *p* < 0.05; ** *p* < 0.01.
