# Supplementary material for: Immunogenicity Evaluating of the Multivalent COVID-19 Inactivated Vaccine against the SARS-CoV-2 Variants
Source: Vaccines (Basel). 2022 Jun 16;10(6):956. doi: 10.3390/vaccines10060956 (PMC9228943; doi:10.3390/vaccines10060956)
Supplement: Supplementary file 1 [file vaccines-10-00956-s001.zip › vaccines-1760368-supplementary.pdf]

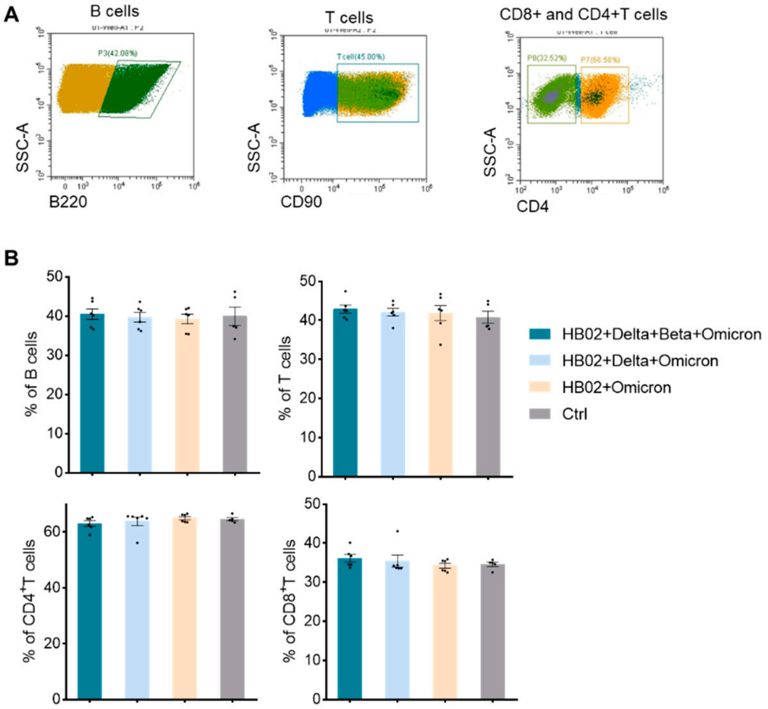

Figure S1. The effect of vaccines on various immune cells. (A) The representative flow cytometry figures of B cells (gated in live CD45+), T cells (gated in live CD45+CD90+), CD8+ T cells (gated in live CD45+CD90+CD4-) and CD4+ T cells (gated in live CD45+CD90+CD4) in spleens are shown. B) Percentages of B cells, T cells, CD4+ T and CD8+T cells in the spleen were analyzed using flow cytometry.

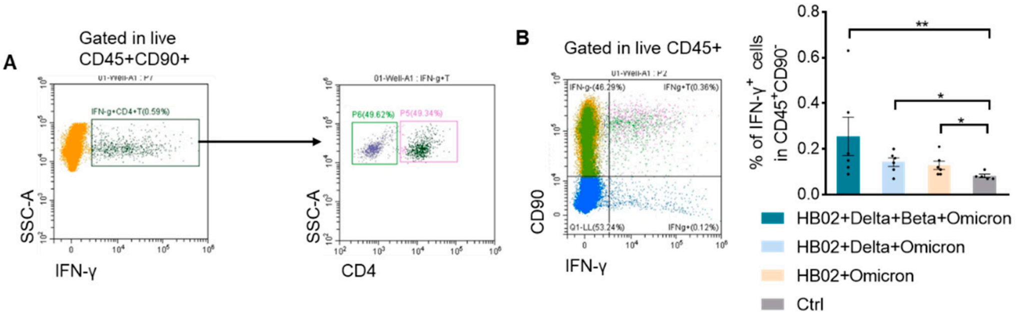

Figure S2. IFN-γ-expressing cell analysis by FACS. (A) The representative flow cytometry figures of IFN-γ+ T cells (gated in live CD45+) in spleens are shown. (B) Percentages of CD90- IFN-γ+cells in the spleen were analyzed using flow cytometry. Error bars represent SEM. *p < 0.05; **p < 0.01.
